# Turning over New Leaves

**Published:** 1888-07-21

**Authors:** 


### TURNING OVER NEW LEAVES.

[Books for Review should be sent to The Editor, The Lodge, Porchester Square, W.]

### Amputations Treated Antiseptically.*

DR. PAGE's pamphlet really deals with the relative mortality of large and small hospitals, and may be taken as a pendant to Mr. Burdett's book on that subject. Mr. Burdett's statistics tended to the conclusion that the hygienic conditions of cottage hospitals were more favourable to recovery after amputations than those of the larger hospitals in towns, although there the operations were performed by men whose names were better known to the world. They also disproved the notion, so often set forth, that in the country patients were allowed to die because doctors lacked the "surgical boldness" to operate when the result was hazardous; whereas in a city hospital the surgeon will almost always risk death as the result of an operation when the only alternative is death without it. Mr. Burdett's list, indeed, included no amputations at the hip, but all other major amputations were represented, and the mortality was 17 per cent. The mortality in the large hospitals of London, Paris, Edinburgh, and Glasgow was then calculated at from 36 to 60 per cent. Dr. Page's paper, however, shows that these favourable results do not inevitably belong to small hospitals; they have been surpassed in the Newcastle Royal Infirmary. During the year 1887 major amputations were performed on 60 patients, of whom only two died—a mortality of 3·3 per cent. Of the deaths, one occurred two hours after the operation—a double amputation required by compound fracture of the thigh and opposite leg —from shock and loss of blood. In the other, the patient, a man of seventy years of age, refused to permit the operation till gangrene had set in, when practically it was too late. It may be suggested that this record is exceptional, and shows an unusually low percentage of deaths, but taking the mortality from amputations in Newcastle Infirmary during the last five years, we find that the number of deaths in 282 cases was only 14, that is, 4·9 per cent. ; while, extending the calculations over ten years, the number of deaths is 31 out of 442 cases, or 7 per cent. This is sufficiently satisfactory, but it is even better to find that it is the mortality of the first five years that brings the death-rate so high. During that time it was 10·6, while during the last five years it was 4·9, which clearly shows the marked improvement in conditions favourable to recovery after operations, especially when we note that only one case died from pyæmia. Mr. Page speaks with somewhat unnecessary humility of the inferiority to be expected in the results of the hospital of a provincial town as compared with London. Most London hospitals would be proud to show results as good as those his statistics prove, and we know for an assured fact that Newcastle in particular can boast of surgeons whom our most distinguished metropolitan doctors might be proud to claim as equal to themselves.

### The Science and Art of Surgery.†

FEW things concern the general public more than the science and art of surgery. It is not merely strange, but almost past belief, that until within the last century or two there was no science of surgery at all, whilst the art was worthy only of contempt. We need not sketch, for the ten thousandth time, the history of the barber-surgeons in this country. The present race of surgeons is as completely separated from them in every particular as the physicians are from the medicine men of savage lands. Surgery is now an art based upon a science. It is in the highest degree a skilled art, and has for its objects the removing of human disabilities, the mitigating of human pains, and the saving of human life. As a science it deals with problems of the profoundest interest to man, both as a material and a moral creature.

It is eminently desirable that the professors of the science and art of surgery should be men of a cultured and comprehensive intelligence, as well as of manipulative skill. Surgery does not consist merely in the performance of cutting or other mechanical operations. It demands the exercise of trained observation, critical judgment, intelligent decision, profound sympathy with suffering and sorrow, and a courageous resolution which surmounts all obstacles until the end to be sought is accomplished. It may readily be conceived that he who aspires to be a great surgeon must have in him the elements of a great man. He who has proved himself a great surgeon and a great teacher of his art, has accomplished something for his fellows which entitles him to their reverence and gratitude.

All the world—that is all the medical world—is familiar with Erichsen's "Surgery" The name of Erichsen is a household word in the medical profession. The present issue is the ninth edition of Erichsen's monumental work. The medical reviewer cannot suppress a sigh when he reflects that however able, brilliant, exhaustive, and complete a medical work may be, it can never take its place in the library, or, in public estimation, as standard literature. To the student of history or poetry, to the readers of Scott and George Eliot, it is alike a sealed book. This, to a writer who has any of the inspiration of genius about him is a most paralysing fact. To think that his ablest reasoning, his most careful deductions, his clear and flowing diction, will only be studied by medical students cramming for examinations, or by country surgeons reading up for operations, is heart-breaking. To think, too, that in fifteen or twenty years, unless successive editors of the right stamp can be found, the labour of years will be relegated to that portion of the bookshelves which has reached the museum level, or may even descend to the deeper depths of the village butter shop, is an anticlimax that makes the reflective man both laugh and weep.

In the meantime Erichsen is here in a new edition, thoroughly revised and brought down to date, well and clearly printed, full of excellent illustrations, most of which are original and many quite new, and "got up" in a style which is worthy of the work and creditable to the publishers. It is not necessary to say that we wish for the new edition a large sale. It is sure to have a large sale. What other work is there in the English tongue which, for fulness, excellence, and completeness is its superior, or even its equal? The book is issued in two thick octavos, and the price is 48*s*.

* "Results of Major Amputations treated Antiseptically in the Royal Infirmary, Newcastle-upon-Tyne, during the year 1887, with remarks." By FREDERICK PAGE, M.D., Honorary Surgeon to the Royal Infirmary.

† "The Science and Art of Surgery." By John Eric Erichsen, F.R.S., LL.D., &c. London: Longmans and Co.

